# SELEX Aptamer Used as a Probe to Detect Circulating Tumor Cells in Peripheral Blood of Pancreatic Cancer Patients

**DOI:** 10.1371/journal.pone.0121920

**Published:** 2015-03-23

**Authors:** Jinqiang Zhang, Shaohua Li, Fang Liu, Lanping Zhou, Ningsheng Shao, Xiaohang Zhao

**Affiliations:** 1 State Key Laboratory of Molecular Oncology, Cancer Institute and Hospital, Chinese Academy of Medical Sciences and Peking Union Medical College, Beijing, China; 2 Department of Biochemistry and Molecular Biology, Beijing Institute of Basic Medical Sciences, Beijing, China; University of Kentucky College of Medicine, UNITED STATES

## Abstract

Many studies have shown that the quantity and dynamics of circulating tumor cells (CTCs) in peripheral blood of patients afflicted with solid tumours have great relevance in therapeutic efficacy and prognosis. Different methods based on various strategies have been developed to isolate and identify CTCs, but their efficacy needs to be improved because of the rarity and complexity of CTCs. This study was designed to examine the possibility of using a SELEX aptamer (BC-15) as a probe to identify rare CTCs out of background nucleated cells. Aptamer BC-15 was selected from a random oligonucleotide library screened against human breast cancer tissue. Fluorescence staining showed that BC-15 had a high affinity for nuclei of human cancer cell lines of various origins as well as CTCs isolated from pancreatic cancer patients, whereas its binding capacity for non-tumor breast epithelial cells and leukocytes was almost undetectable. BC-15^+^/CD45^-^ cells in cancer patient blood were also found to be cytokeratins 18-positive and aneuploid by immunofluorescence staining and fluorescent in situ hybridization, respectively. Finally, the aptamer method was compared with the well-established anti-cytokeratin method using 15 pancreatic cancer patient blood samples, and enumeration indicated no difference between these two methods. Our study establishes a novel way to identify CTCs by using a synthetic aptamer probe. This new approach is comparable with the anti-cytokeratin-based CTC identification method.

## Introduction

Circulating tumor cells (CTCs) have been found in the peripheral blood of patients afflicted with all major solid carcinomas[1] and their quantity and fluctuation in cancer patient blood correlated with tumor development, therapeutic efficacy, tumor recurrence, and long-term prognosis[2–4]. In addition, CTC detection also provides a possible way to monitor patient response to certain anti-cancer therapies[5, 6]. Many techniques have been developed to identify CTCs in patients with different types of cancer[7]. The predominant strategy exploits the epithelial origin of CTCs to capture and identify them using antibodies that target epithelial markers such as epithelial cellular adhesion molecule (EpCAM) and cytokeratin (CK)[2]. However, some CTCs may lose their epithelial characteristics because of the epithelia-mesenchymal transition (EMT) and thus cannot be detected by these antibodies[8]. Furthermore, these methods have difficulty discriminating malignant cells from benign cells that express epithelial markers. The development of novel and more effective approaches for CTCs detection is urgently needed.

Aptamers represent a group of single-stranded nucleic acid fragments that were screened against specific targets from a random synthetic nucleic acid library by the method of systematic evolution of ligands by exponential enrichment (SELEX)[9, 10]. One can even get aptamers, which specifically binding to a molecule without knowing its characteristics. Aptamers can bind to targets with high affinity via specific structural regions that are induced by sequence-dependent folds[11]. This technique has been used for many purposes, including protein inhibitor design, molecular detection, and therapeutic drug and antibody replacement[7]. In previous study[12], we have identified a tumor specific aptamer BC-15 using a new *in situ* tissue slide-based SELEX strategy. Aptamer BC-15 has also been proved to bind to multiple cancer cells of various origins with high specificity. Through streptavidin magnetic beads mediated affinity purification assay followed by mass spectrometry identification and western blot confirmation, the target of BC-15 was characterized to be heterogeneous nuclear ribonucleoprotein A1 (hnRNP A1). The hnRNP family proteins play important roles in biogenesis and transport of messenger RNAs. Up-regulation of hnRNPs usually precedes morphological differentiation and is considered a good biomarker in the early stages of cancer development[13]. Enhanced amounts of hnRNP A1 has been reported in many cancer tissues including breast, and small cell lung, ovarian, colorectal carcinoma, and pancreatic cancer with a location that is mainly nuclear [13–16]. High levels of hnRNP A1 expression was also proved in pancreatic tumor cell lines, whereas in normal primary pancreatic cells hnRNP expression was undetectable. These results strongly suggest that hnRNP could be a good candidate for diagnosis of pancreatic cancer[13]. Therefore, aptamer BC-15 could also be used as diagnosis biomarker for multiple types of cancer, including pancreatic cancer due to its high specific affinity to hnRNA A1. Here, we reported the feasibility of using an aptamer BC-15 as a probe to identify CTCs in the peripheral blood of patients with pancreatic cancer.

## Material and Methods

### Blood specimen collection

This study was approved by the ethics review committees of Cancer Hospital of Chinese Academy of Medical Sciences, and informed written consents were obtained from all the pancreatic cancer patients and healthy donors. A total of 30 blood samples were collected, including 15 samples taken from pancreatic cancer patients and 15 samples from healthy donors. For each patient or healthy donor, peripheral blood (7.5ml) was drawn from the median cubital vein into acid citrate dextrose vacutainer tubes (BD Diagnostics, Franklin Lakes, NJ) after discarding the first 3 ml of blood to avoid epithelial cell contamination during venipuncture. All samples were maintained at room temperature and processed within 12 h after collection. To avoid bias, samples were blindly processed by different persons.

### SELEX procedure

A new *in situ* tissue slide-based SELEX strategy was employed to select high-affinity aptamers. Formalin-fixed, paraffin-embedded breast infiltrating ductal carcinomas and adjacent normal tissue from the same patient were used as the SELEX target and control respectively. After 12 rounds of screening, BC-15 was selected for its high affinity for tumor tissue specifically, which was also verified using cell lines. The entire SELEX screening process has been described in our previous study[12]. The aptamer probe BC-15 (5’-GCAATGGTACGGTACTTCCTGTGGCGAGGTAGGTGGGGTGTGTGTGTATCCAAAAGTGCACGCTACTTTGCTAA-3’) was synthesized and labelled with fluorescein isothiocyanate (FITC) on the 5’ end (Life Technologies, Grand Island, NY, USA), and a mixture of random sequences labelled the same way acted as a control probe.

### Cell culture

Human cancer PL-45 (ATCC CRL-2558, pancreatic adenocarcinoma), MCF-7 (ATCC HTB22, breast adenocarcinoma), A549 (ATCC CCL185, lung carcinoma), MDA-MB-231 (ATCC HTB-26, metastatic breast adenocarcinoma), HT-29 (ATCC HTB-38, colorectal adenocarcinoma) and MCF10A (ATCC CRL10317, breast fibrocystic disease) cell lines were purchased from American Type Culture Collection (Rockville, MD, USA). All cells except MCF10A were cultured in Dulbecco’s Modified Eagle’s Medium (DMEM) supplemented with 10% fetal bovine serum (Gibco, Grand Island, NY, USA). MCF10A was cultured in DMEM supplemented with 20% fetal bovine serum, 20ng/ml human epidermal growth factor (EGF), 10μg/ml insulin, and 0.5μg/ml hydrocortisone. All cells were incubated in a humidified atmosphere with 5% CO_2_ at 37°C. For fluorescence staining, overnight cultured cells were fixed in methanol at −20°C for 2 h and washed with phosphate-buffered saline (PBS, pH 7.4), and then cells were stained with aptamer as described in the following section.

### Enrichment and identification of CTCs

The CTCs enrichment method was similar to the previously published methods[17]. Briefly, 7.5 ml of peripheral blood collected in a vacutainer tube was washed with PBS once, and then red blood cells were lysed with lysis buffer (150 mM NH_4_Cl, 10mM NaHCO_3_, and 0.1mM EDTA). The reaction mixture was spun down at 300 × *g* for 5 min at room temperature. The resulting cell pellet was resuspended in PBS and subsequently incubated with 0.1 ml of anti-CD45- antibody coated magnetic beads (Miltenyi Biotec, Bergisch Gladbach, Germany) for 30 min at room temperature, followed by separation using a magnetic stand (Promega, Madison, WI, USA). Supernatants were transferred into a centrifuge tube followed by spinning at 500 × *g* for 3 min at room temperature. Each cell pellet was resuspended in PBS and dropped equally onto two slides (Thermo Fisher Scientific, Waltham, MA, USA), air dried, and then subjected to staining.

CTCs were identification by BC-15 aptamer or anti-CK staining. For BC-15, slides were fixed with methanol for 30 min at -20°C followed by penetration with 0.1% (w/v) Tween 20 in PBS at room temperature for 15 min. After blocking with buffer (0.1mg/ml tRNA, 0.1mg/ml salmon sperm DNA, 1% BSA and 0.02% (w/v) Tween-20 in PBS) at 37°C for 20 min, slides were incubated with 10ug/ml FITC-labelled BC-15 in blocking buffer at 37°C for 1 h. Anti-CK CTC identification was conducted as described in many studies [2, 7, 18]. Briefly, slides were fixed with paraformaldehyde for 40 min followed by 0.1% (w/v) Triton X-100 penetration and 2% BSA blocking, and then incubated with FITC-labelled anti-CK (Miltenyi Biotec; 1:1000 dilution in PBS), which recognises CKs 8, 18, and 19, at room temperature for 1 h. Alexa 594 labelled anti-CD45 (Miltenyi Biotec; 1:1000 dilution in PBS) was used to co-staining experiments with BC-15 or anti-CK as a negative selection signal. After incubation, slides were washed with PBS and then mounted with 4’-6-diamidino-2-phenylindole (DAPI)-containing mounting medium (Vector Labs, Burlingame, CA, USA). Then slides were subjected to analysis with a Nikon Eclipse 80i fluorescence microscope. Cells that met the following criteria were considered as CTCs: cell size >4μm in diameter, intact with round-to-oval morphology with a DAPI-stained nucleus, and positive for CK or BC-15 staining and negative for CD45[18].

### Aneuploid chromosome analysis using fluorescence *in situ* hybridisation (FISH)

Chromosome enumeration probes (CEP) 8 (Vysis, Des Plaines, IL, USA) was denatured in hybridization buffer (0.15 M sodium chloride, 0.015 M sodium citrate, 70% formamide) at 73°C for 5min. The probe was then incubated with a slide in a humidified chamber at 42°C overnight followed by washing with 0.3% (w/v) NP-40 in 0.4x saline-sodium citrate (SSC; 1x SSC containing 150 mM NaCl and 15 mM Na_3_Citrate) at 73°C for 2 min and with 2x SSC/0.1% NP-40 at room temperature for 30 s. After briefly drying, slides were analyzed after mounting with DAPI mounting medium (Vector Labs) under fluorescence microscope.

### Statistical analysis

Difference between these two identification methods was evaluated by SPSS 19 (IBM, Armonk, NY, USA) software with Wilcoxon Signed Ranks test and the correlation between BC-15 and anti-cytokeratin results was assessed by the nonparametric Spearman’s rho value. A *P*-value of <0.05 was consider as the statistical significance.

## Results

### BC-15 binds to the nucleus of human tumor cells specifically

**Fig 1:**
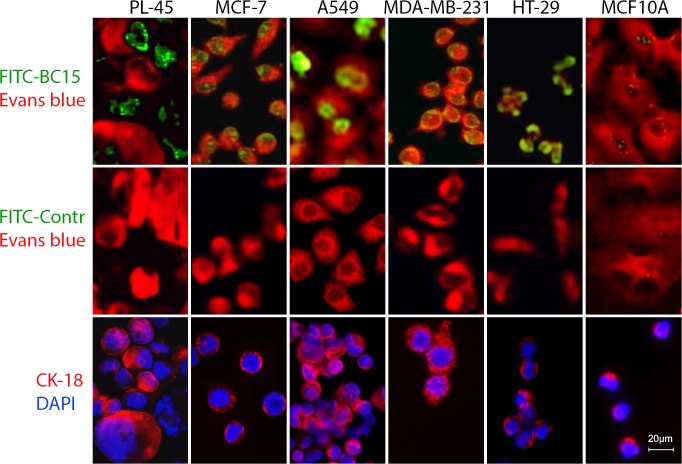
BC-15 binds to various kinds of human cancer cells. Cells were stained with FITC-labelled BC-15 aptamer (Upper row) or FITC-labelled random control aptamer (Middle row, green). All the cells were counter stained with 0.25% Evans blue for 10 min to reveal whole-cell morphology (red). Lower row show CK immunofluorescence staining of different types of cancer cells. These cultured cells were trypsinized, dropped onto the coated glass slides, followed by staining with Alexa 594 labelled anti-CK18 antibody, and counter stained with DAPI. PL45, pancreatic adenocarcinoma cell; MCF-7, breast cancer cell; A549, lung carcinoma cell; MDA-MB-231, breast cancer cell; HT-29, colon adenocarcinoma cell; MCF10A, non-tumourigenic immortalised mammary epithelial cells. Scale bar, 20μm.

Our previous study shows that BC-15 can bind to breast cancer cells and tissues with high affinity, whereas its binding capacity for non-tumorigenic cells and normal breast tissues is much lower; in addition to fluorescence staining, flow cytometric confirmed that BC-15 has a high affinity for tumor cells [12]. To validate the binding of BC-15 to tumor cells other than breast cancer cells, a set of human cancer cells was stained with BC-15 (Fig. 1). BC-15 signals accumulated mainly in nuclei of cancer cells but were much lower in immortalised breast epithelial MCF10A cells. No positive signals were observed in any cells when staining with a random control aptamer probe. This observations suggested that BC-15 exclusively binds to nuclei of cancer cells.

### BC-15^+^ cells in patient peripheral blood are also CK-positive

**Fig 2:**
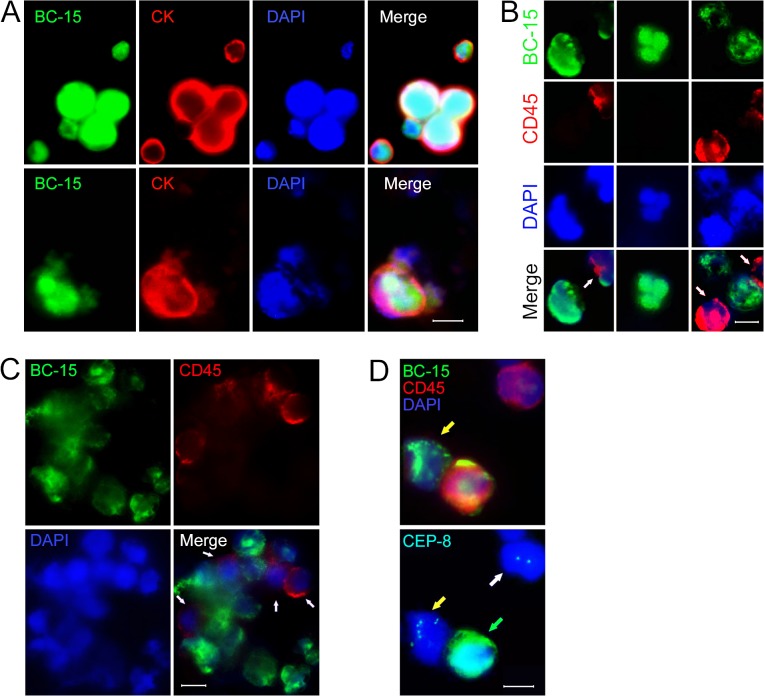
CTCs identified in pancreatic cancer patient peripheral blood by BC-15. (A) BC-15^+^ (green) and CK18^+^ (red) signals coincided in both PL-45 cells (Upper row) and CTCs from pancreatic cancer patients (Lower row). (B) Morphology of dispersed CTCs from pancreatic cancer patients identified by immunofluorescence staining. CTCs were demonstrated as CD45^–^cells with BC-15^+^ nuclei (green) and the arrows in the bottom images of panel A indicat the white blood cells (CD45^+^, red). (C) Clustered CTCs recognized by BC-15 in blood samples from pancreatic cancer patients. White arrows indicate leukocytes (CD45^+^). (D) Immunofluorescence of a BC-15^+^ cells (noted by yellow arrow) was quenched and the same slide was subjected to FISH using a CEP8 probe. BC-15^+^ cell had a chromosome 8 aberration (7 copies), whereas the background leukocyte was diploid for chromosome 8 (white arrow). The cell indicated by green arrow was not classified as CTC because of its positivity for both BC-15 and CD45. Scale bars, 10μm.

Given that BC-15 has a high and specific affinity to CK-positive cancer cells of diverse origins (Fig. 1 and Fig. 2A), we stained patient samples with the BC-15. The morphology of BC-15^+^ cells is shown in Fig. 2B. BC-15 signals were observed exclusively in cell nuclei, which is similar to previously described patterns in human cancer cells and tissues. Moreover, the vast majority of BC-15^+^ cells were CD45^–^, and BC-15 binding to leukocytes (CD45^+^) was not detected (Fig. 2B and 2C). Pancreatic cancer PL-45 cells and some pancreatic cancer patient samples, which stained positively with anti-CK, were then re-stained by BC-15. As shown in Fig. 2B, BC-15 and CK signals coincided well in both PL-45 cells (upper row) and CTCs from pancreatic cancer patient samples (lower row). Next, FISH experiments using CEP8 were performed to verify the aneuploid character of BC-15^+^ cells. As shown in Fig. 2D, BC-15^+^/CD45^-^ cells were abnormal in chromosome 8 enumeration, whereas the white blood cells (CD45^+^) in the background was diploid for chromosome 8. Besides single and doublet CTCs, tumor cell-lymphocyte mixed clusters were also detected with BC-15 (Fig. 2C); such clusters have been proven to be a more accurate prognostic factor for metastasis as compared with the presence of single circulating tumor cells[17].

### BC-15 has the similar efficacy as anti-CK8/18/19 for identifying CTCs

Anti-CK-based immunostaining is an established method to detect CTCs in many systems and shows relative satisfactory consistency and repeatability in various kinds of cancers types[19]. The malignant characteristics of CK^+^ cells have also been proven by FISH with various CEP probes. To compare the CTC identification capacity of BC-15 with that of anti-CK methods, 15 peripheral blood samples from pancreatic cancer patients (Table 1) and 15 from healthy donors were collected. The cells enriched from these samples were divided equally and stained side by side using these two methods. Neither method detected positive cells in the samples from healthy donors. Of the 15 patients samples, 12 (80.0%) had positive cells according to the anti-CK method, whereas the positive rate was 73.3% (11/15) using BC-15 staining. The average number of positive cells detected by anti-CK or the BC-15 staining was 25.7 and 19.1 per sample respectively. No statistic difference was found between the CTC enumeration results of the two methods by Wilcoxon Signed Ranks test (*P* = 0.699) and there was significant correlation between the results of these two different CTC detection methods by Spearman's rank correlation test (Spearman’s rho = 0.810, *P* < 0.01).

**Table 1:** CTCs detection in patient samples using BC-15 aptamer versus anti-CK.

Patient ID	Sex	Age	Diagnosis^a^ (Methods)^b^	CTC number	
				BC-15	Anti-CK
317h	M	68	PDA (S)	0	1
323h	M	54	PDA (S)	1	0
324h	M	67	PDA (S)	0	0
325q	M	59	PDA (S)	0	0
329q	M	56	PDA (S)	30	2
330q	F	51	SPT (S)	12	4
332q	F	56	PDA (F)	50	28
332h	F	56	PDA (F)	100	108
334q	F	59	PDA (F)	3	4
334h	F	59	PDA (F)	23	126
335q	M	35	Malignant (PET)	10	5
337q	M	78	PDA (F)	4	3
339q	M	53	PDA (F)	45	68
339h	M	53	PDA (F)	0	1
341q	F	68	PDA (S)	8	36

a: PDA, pancreatic ductal adenocarcinoma; SPT, solid pseudopapillary tumor of the pancreas; PET, pancreatic endocrine tumor.

b: S, surgical biopsy; F, fine needle aspiration; PET, positron emission tomography.

## Discussion

As a surrogate of primary or metastatic tumors, CTCs are showing increasing importance both in cancer research and clinical practice, and CTC detection has gained intensified interest within the cancer research community. In the present study, we successfully established a CTC detection system combining a leukocyte depletion enrichment strategy and BC-15 aptamer identification. This assertion is supported by the following observations: (1) BC-15 recognised various kinds of tumor cells specifically, and non-tumorigenic epithelial cells and leukocytes were negative for BC-15 staining; (2) BC-15 had the same staining pattern in BC-15^+^/CD45^-^ cells enriched from pancreatic cancer patient blood as in cancer cells and tumor tissues; (3) BC-15^+^ cells in cancer patients blood were CK^+^[20]; (4) BC-15^+^/CD45^-^ cells in blood from cancer patients showed aneuploidy, a cytogenetic abnormality typical of malignant cells; and (5) the CTC identification efficacy of BC-15 was equivalent to that of anti-CK methods.

Because of their heterogeneic characteristics and the mesenchymal-epithelial transition in circulation, CTCs cannot be fully enumerated by antibodies that target epithelial markers (e.g. anti- CK)[21]. However, aptamers could be developed to recognise subtle characteristics and/or low-immunogenic molecules of malignant cells. Our preliminary results presented here demonstrate that it is feasible to use an aptamer as a CTC detection probe and also provide perspectives that would make CTC detection more customised and personalised. Although our pilot results show no improvement on efficacy of CTC identification compared with antibody-based methods, the aptamer probe holds great promise for CTCs detection (both isolation and identification) owing to its capacity to bind to various types of molecular targets, high affinity, well defined synthesis/modification process, stability and uniformity. Furthermore, based on the well-developed cell/tissue-SELEX technique, it is possible to generate particular aptamers with high stability by using tumor cells or tumor tissues from a given patient as selecting targets[22]. These personalised aptamers can partly overcome the weakness raised by uniform antibodies[23] and can be used as the guidance molecular of targeting therapy or used to monitor therapeutic response and anticipate long term prognosis [22].

## References

[1] Allard WJ, Matera J, Miller MC, Repollet M, Connelly MC, Rao C, et al Tumor cells circulate in the peripheral blood of all major carcinomas but not in healthy subjects or patients with nonmalignant diseases. Clin Cancer Res. 2004;10(20):6897–6904. 15501967 10.1158/1078-0432.CCR-04-0378

[2] Cristofanilli M, Budd GT, Ellis MJ, Stopeck A, Matera J, Miller MC, et al Circulating tumor cells, disease progression, and survival in metastatic breast cancer. N Engl J Med. 2004;351(8):781–791. 15317891 10.1056/NEJMoa040766

[3] Moreno JG, Miller MC, Gross S, Allard WJ, Gomella LG, Terstappen LW. Circulating tumor cells predict survival in patients with metastatic prostate cancer. Urology. 2005;65(4):713–718. 15833514 10.1016/j.urology.2004.11.006

[4] Zippelius A, Pantel K. RT-PCR-based detection of occult disseminated tumor cells in peripheral blood and bone marrow of patients with solid tumors. An overview. Ann N Y Acad Sci. 2000;906:110–1123. 10818606 10.1111/j.1749-6632.2000.tb06600.x

[5] Fehm T, Sagalowsky A, Clifford E, Beitsch P, Saboorian H, Euhus D, et al Cytogenetic evidence that circulating epithelial cells in patients with carcinoma are malignant. Clin Cancer Res. 2002;8(7):2073–2084. 12114406

[6] Meng S, Tripathy D, Shete S, Ashfaq R, Saboorian H, Haley B, et al uPAR and HER-2 gene status in individual breast cancer cells from blood and tissues. Proc Natl Acad Sci U S A. 2006;103(46):17361–17365. 17079488 10.1073/pnas.0608113103 PMC1838539

[7] Greene BT, Hughes AD, King MR. Circulating tumor cells: the substrate of personalized medicine? Front Oncol. 2012;2:69 10.3389/fonc.2012.00069 22783545 PMC3387782

[8] Yu M, Bardia A, Wittner BS, Stott SL, Smas ME, Ting DT, et al Circulating breast tumor cells exhibit dynamic changes in epithelial and mesenchymal composition. Science. 2013;339(6119):580–584. 10.1126/science.1228522 23372014 PMC3760262

[9] Tuerk C, Gold L. Systematic evolution of ligands by exponential enrichment: RNA ligands to bacteriophage T4 DNA polymerase. Science. 1990;249(4968):505–510. 2200121 10.1126/science.2200121

[10] Ye M, Hu J, Peng M, Liu J, Liu J, Liu H, et al Generating Aptamers by Cell-SELEX for Applications in Molecular Medicine. Int J Mol Sci. 2012; 13(3):3341–3353. 10.3390/ijms13033341 22489154 PMC3317715

[11] Bridonneau P, Chang YF, Buvoli AV, O'Connell D, Parma D. Site-directed selection of oligonucleotide antagonists by competitive elution. Antisense Nucleic Acid Drug Dev. 1999; 9(1):1–11. 10192284 10.1089/oli.1.1999.9.1

[12] Li S, Xu H, Ding H, Huang Y, Cao X, Yang G, et al Identification of an aptamer targeting hnRNP A1 by tissue slide-based SELEX. J Pathol. 2009; 218(3):327–336. 10.1002/path.2543 19291713

[13] Yan-Sanders Y, Hammons GJ, Lyn-Cook BD. Increased expression of heterogeneous nuclear ribonucleoprotein A2/B1 (hnRNP) in pancreatic tissue from smokers and pancreatic tumor cells. Cancer Lett. 2002; 183(2):215–220. 12065097 10.1016/s0304-3835(02)00168-4

[14] Patry C, Bouchard L, Labrecque P, Gendron D, Lemieux B, Toutant J, et al Small interfering RNA-mediated reduction in heterogeneous nuclear ribonucleoparticule A1/A2 proteins induces apoptosis in human cancer cells but not in normal mortal cell lines. Cancer Res. 2003; 63(22):7679–7688. 14633690

[15] Zerbe LK, Pino I, Pio R, Cosper PF, Dwyer-Nield LD, Meyer AM, et al Relative amounts of antagonistic splicing factors, hnRNP A1 and ASF/SF2, change during neoplastic lung growth: implications for pre-mRNA processing. Mol Carcinog. 2004; 41(4):187–196. 15390079 10.1002/mc.20053

[16] Ushigome M, Ubagai T, Fukuda H, Tsuchiya N, Sugimura T, Takatsuka J, et al Up-regulation of hnRNP A1 gene in sporadic human colorectal cancers. Int J Oncol. 2005; 26(3):635–640. 15703818

[17] Molnar B, Ladanyi A, Tanko L, Sreter L, Tulassay Z. Circulating tumor cell clusters in the peripheral blood of colorectal cancer patients. Clin Cancer Res. 2001; 7(12):4080–4085. 11751505

[18] Wu C, Hao H, Li L, Zhou X, Guo Z, Zhang L, et al Preliminary investigation of the clinical significance of detecting circulating tumor cells enriched from lung cancer patients. J Thorac Oncol. 2009; 4(1):30–36. 10.1097/JTO.0b013e3181914125 19096303

[19] Balic M, Lin H, Williams A, Datar RH, Cote RJ. Progress in circulating tumor cell capture and analysis: implications for cancer management. Expert Rev Mol Diagn. 2012; 12(3):303–312. 10.1586/erm.12.12 22468820 PMC3391569

[20] Pantel K, Brakenhoff RH, Brandt B. Detection, clinical relevance and specific biological properties of disseminating tumour cells. Nat Rev Cancer. 2008; 8(5):329–340. 10.1038/nrc2375 18404148

[21] Ksiazkiewicz M, Markiewicz A, Zaczek AJ. Epithelial-mesenchymal transition: a hallmark in metastasis formation linking circulating tumor cells and cancer stem cells. Pathobiology. 2012; 79(4):195–208. 10.1159/000337106 22488297

[22] Song KM, Lee S, Ban C. Aptamers and their biological applications. Sensors (Basel). 2012; 12(1):612–631. 10.3390/s120100612 22368488 PMC3279232

[23] Kunii T, Ogura S, Mie M, Kobatake E. Selection of DNA aptamers recognizing small cell lung cancer using living cell-SELEX. Analyst. 2011; 136(7):1310–1312. 10.1039/c0an00962h 21321690

